# Acute Onset Budd-Chiari Syndrome in the Postpartum Period: A Case of Missed Diagnosis Leading to Rapid Deterioration

**DOI:** 10.7759/cureus.73284

**Published:** 2024-11-08

**Authors:** Malalai Alami, Bibi Sarah Yousofzai, Salman Shafiq, Rida Mehmood, Muhammad Subhan, Ruqiya Bibi

**Affiliations:** 1 Obstetrics and Gynaecology, Rabia Balkhi Hospital, Kabul, AFG; 2 Obstetrics and Gynaecology, Be Team Cure International Hospital, Kabul, AFG; 3 Internal Medicine, Iqbal Medical College, Lahore, PAK; 4 Medicine, Rawalpindi Medical University, Islamabad, PAK; 5 Medicine, Allama Iqbal Medical College, Lahore, PAK

**Keywords:** budd-chiari syndrome, hellp syndrome, liver insufficiency, postpartum complications, venous thrombosis

## Abstract

Budd-Chiari syndrome (BCS), a rare hepatic vein obstruction condition, poses significant risks during gestation and the postpartum period. We present the case of a 30-year-old primigravida at 32 weeks gestation admitted with weakness and lethargy, which was diagnosed with impending uterine rupture, HELLP syndrome, and intrauterine fetal demise. An emergency cesarean section was performed, delivering a stillborn baby and uncovering 700 milliliters of blood clots in her abdominal cavity. Postoperatively, she experienced severe headaches, chest tightness, epigastric pain, nausea, vomiting, persistent fever, and abdominal distension, all of which were severe and persistent. Although treatment for sepsis and HELLP syndrome was initiated, BCS was eventually confirmed through ultrasound and Doppler sonography, though advanced imaging techniques were limited. Aggressive treatment with antibiotics and supportive care could not prevent worsening liver function, leading to severe bradycardia and death within five days. This case underscores the necessity of prompt recognition and multidisciplinary management of BCS among pregnant and postpartum women to improve outcomes in this rare yet critical condition.

## Introduction

Budd-Chiari syndrome (BCS), caused by obstruction of the hepatic vein, affects approximately one person out of every million [1]. Its incidence during pregnancy and postpartum has been recognized since 1899, when the first case was reported immediately post-childbirth, likely due to the hypercoagulable state associated with pregnancy [2]. BCS affects two people per million in Japan and is typically caused by thrombotic or non-thrombotic hepatic vein obstruction, leading to hepatomegaly and splenomegaly [2-4]. About 75% of primary BCS cases can be attributed to thrombosis in the hepatic veins [2,3]. Hematologic conditions like sickle cell disease, thrombocythemia, thrombophilia, myelodysplastic syndrome, polycythemia vera, and paroxysmal nocturnal hemoglobinuria account for a significant portion, with the latter contributing to 23% of cases [3,4]. Autoimmune disorders, such as lupus erythematosus and antiphospholipid antibody syndrome, are also linked to BCS [4,5]. These disorders can lead to the formation of blood clots in the hepatic veins, contributing to the development of BCS [3,4]. Additionally, neoplasms are responsible for 13% of cases, while hormonal factors, including contraceptive pill use (23%) and hormone replacement therapy (2%), further contribute to the development of the condition [2,3]. Secondary BCS accounts for about 25% of cases and is often associated with hepatic hemangiomas [1,2]. Risk factors include genetic disorders, inflammatory conditions, and immunosuppression from medications or conditions like Bechet's syndrome [1,2]. Liver cirrhosis and cancerous growths in the liver tissue also contribute to secondary BCS [3,4]. Infections such as tuberculosis, syphilis, and aspergillosis have been identified as potential triggers [1-4]. Despite these known causes, approximately 33% of cases remain idiopathic, with no identifiable underlying condition [1-3].

Differential diagnoses could include ascites, hepatic arterial lesions, and postpartum polycythemia vera [3]. Diagnosing BCS involves an evaluation of the patient's history, symptoms, physical examination, and specific diagnostic tests [3]. The clinical manifestations of BCS can vary, ranging from chronic, slowly progressing symptoms such as abdominal pain, hepatomegaly, and ascites to acute, more severe presentations, including sudden liver failure, jaundice, and life-threatening complications [4-6]. The initial symptoms of BCS may include mild pain, bloating, nausea, vomiting, fatigue, and right upper quadrant discomfort [3,4]. As the condition progresses, patients can develop more severe complications such as jaundice, ascites, and hepatosplenomegaly [4,5]. Additionally, advanced cases may lead to conditions like liver fibrosis, portal hypertension, and the formation of esophageal varices, as well as signs like lower limb edema [6,7]. Pregnant women with chronic liver conditions, including BCS, are at greater risk for anemia and related complications during gestation [5,6]. These complications, which are particularly associated with BCS and other severe liver conditions, include miscarriage, preterm birth, intrauterine growth restriction (IUGR), fetal death, and preeclampsia [6,7]. However, some of these complications, such as anemia and preeclampsia, may also occur with other chronic liver conditions [7,8].

Pregnancy-specific diagnostic criteria for BCS should include an assessment of risk factors such as age, a history of smoking, and the use of prothrombotic medications before gestation [4,5]. Additionally, complications from conditions like portal hypertension, as well as any previous treatments, should be considered [5,6]. Diagnostic tools, including clinical assessments, laboratory tests, and imaging techniques such as Doppler ultrasonography, angiography, venography, or MRI scans, are crucial in confirming the diagnosis and assessing disease severity [5]. Liver biopsy is used when diagnostic imaging is insufficient [5]. Hence, results demonstrating dilation and central sinusoid congestion are vital in diagnosing non-thrombotic hepatic vein obstruction [5].

Treatment depends on the severity of venous obstruction and liver damage, from anticoagulants and diuretics to angioplasty or liver transplantation [5,6]. Delay in treatment significantly increases mortality rates; survival rates range from 38% to 87% after portosystemic shunt placement [5]. Anticoagulant medications used during pregnancy can increase the risk of bleeding during and after childbirth [7,8]. This often requires blood transfusions and close monitoring [8,9]. In addition, thromboprophylaxis therapy is needed to prevent blood clots, and some cases may require neonatal intensive care services for the newborn [9,10].

## Case presentation

At 32 weeks gestation, a 30-year-old primigravida woman presented to a hospital with weakness and lethargy. Due to the failure to progress, concerns arose about intrauterine fetal demise (IUFD) and the risk of imminent rupture. As a result, she underwent a cesarean section, delivering a stillborn baby weighing 3000 grams. Spinal anesthesia was given during surgery, during which yellow-colored free ascitic fluid was seen accumulating within her abdomen while approximately 700 milliliters of blood clots were extracted before postoperatively receiving intravenous antibiotics for her recovery. Postoperatively, the patient experienced headaches, chest tightness, epigastric pain, nausea, vomiting, and weakness that suddenly worsened. On examination, she appeared distressed and anxious with a blood pressure of 80/60mmHg, a heart rate of 144 beats per minute with a regular rhythm, oxygen saturation at 81%, and a temperature of 38.3°C. Physical examination revealed icteric sclerae, respiratory distress, as well as a distended abdomen with tenderness in the epigastric area but without signs of peritonitis; a contracted uterus, but no vaginal bleeding was present-leading to suspected sepsis, which led to transference into the intensive care unit (ICU). At admission into the ICU, her vital signs included a blood pressure of 90/70 mmHg, a heart rate between 140 and 150 beats per minute, a temperature between 37.5°C and 37.8°C, and normal urine output. After 2 days, the patient began experiencing fever, respiratory distress, headache, abdominal distension, ascites, and lower extremity edema. She was later diagnosed provisionally with HELLP syndrome, sepsis postpartum infection, and hepatic encephalopathy. Clinical findings, including persistent fever, ascites, and hepatic disturbances, indicated that BCS should be the primary diagnosis and excluded HELLP syndrome as a possible differential.

**Figure 1:**
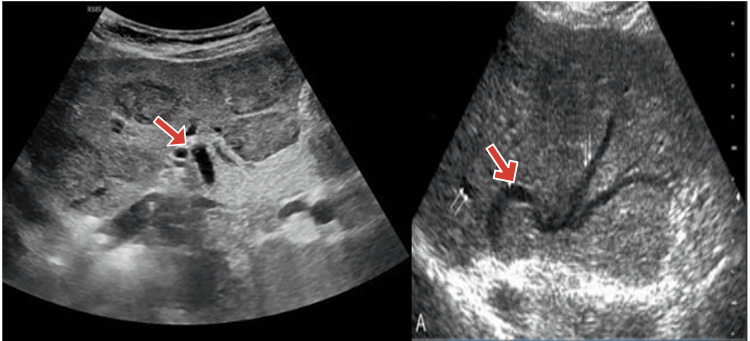
Ultrasound showing Liver With Dilated Biliary Ducts This part of the ultrasound shows a transverse section of the liver and biliary tree. The dilated bile ducts can be visualized, which is suggestive of biliary obstruction. The intrahepatic bile ducts are prominently shown by the red arrows.

A complete blood count (CBC), biochemistry, urine analysis, stool examination, and vaginal swab for culture and identification of an infectious source were performed. Urinalysis revealed WBC 5-7, RBC 4-2, and epithelial cells 10-7. Table 1 presents the patient's laboratory investigations, highlighting significant abnormalities in the coagulation profile and liver function tests. Notably, the platelet count, prothrombin time (PT), activated partial thromboplastin time (APTT), and international normalized ratio (INR) are severely deranged, indicating coagulopathy. Additionally, elevated liver enzymes, total bilirubin, and D-dimer levels reflect hepatic dysfunction and systemic involvement. Ultrasonography confirmed the diagnosis of BCS with Doppler color sonography, showing reduced Doppler flow within hepatic veins due to thrombosis; however, MRI and endoscopic evaluations were deemed unfeasible due to the hospital's facilities. Figure 1 shows the dilated biliary ducts on ultrasound.

**Table 1:** Laboratory Findings in a Pregnant Patient With Budd-Chiari Syndrome

Parameter	Patient’s Results	Normal Range
Blood Group	BRh+	-
Hemoglobin (gr/dL)	11.2	12.0 - 15.5 g/dL (Female)
White Blood Cells (WBC) (/mm³)	19,000	4,000 - 11,000 /mm³
Red Blood Cells (RBC) (million/mm³)	6.8	4.2 - 5.4 million/mm³ (Female)
Platelet Count (/mm³)	48,000	150,000 - 450,000 /mm³
Erythrocyte Sedimentation Rate (ESR) (mm/hr)	19	0 - 20 mm/hr (Female)
Alanine Aminotransferase (ALT) (IU/L)	103	7 - 56 IU/L
Aspartate Transaminase (AST) (U/L)	110	10 - 40 U/L
Total Bilirubin (mg/dL)	8.9	0.1 - 1.2 mg/dL
Direct Bilirubin (mg/dL)	4.4	0.0 - 0.4 mg/dL
Indirect Bilirubin (mg/dL)	4.5	0.1 - 1.2 mg/dL
Lactate Dehydrogenase (LDH) (U/L)	389	140 - 280 U/L
Alkaline Phosphatase (ALP) (U/L)	201	44 - 147 U/L
Gamma Glutamyl Transferase (GGT) (U/L)	74	9 - 48 U/L
Total Proteins (g/dL)	5.8	6.0 - 8.3 g/dL
Creatinine (mg/dL)	2.7	0.6 - 1.2 mg/dL
Urea (mg/dL)	110	7 - 20 mg/dL
Fasting Blood Sugar (FBS) (mg/dL)	60	70 - 100 mg/dL
Prothrombin Time (PT) (sec)	112	11 - 13.5 sec
Activated Partial Thromboplastin Time (APTT) (sec)	90	30 - 40 sec
INR	9.3	0.8 - 1.1
D-dimer (μg/L)	683.57	< 500 μg/L

The fever persisted despite receiving antibiotics Imipenem (1g IV every 8 hours) and Linezolid (600 mg IV every 12 hours), administered intravenously for a duration of five days. The patient's platelet count gradually decreased to 48,000/mm³ while her white blood cell count elevated. Elevated alkaline phosphatase, bilirubin, lactate dehydrogenase (LDH), blood urea, and creatinine levels indicated worsening liver function, which, along with hypoglycemia, increased ascites, and hepatic insufficiency, strongly suggested the progression of hepatic failure associated with BCS due to hepatic venous outflow obstruction. Despite resuscitation efforts, her condition rapidly deteriorated, leading to her death within five days of admission.

## Discussion

BCS is a rare but severe condition characterized by thrombotic obstruction of hepatic venous outflow [1,2]. The condition presents significant challenges during pregnancy and the postpartum period, as these phases are associated with an elevated risk of thrombotic events [1-3]. Venous thromboembolism, including BCS, is a significant cause of maternal mortality, and its peak incidence is usually 6-12 weeks postpartum [4,5]. Global prevalence rates for BCS during pregnancy and postpartum vary widely from 0.05% to 1.4% [5,6]. Recent systematic reviews found an average 6.8% incidence during gestation and reported rates as high as 12.1% [7,8]. Geographically specific prevalence levels include 0.56 cases per million in Denmark compared to 2.4 cases per million in Japan [9,10]. When diagnosing BCS in the postpartum period, it is crucial to distinguish it from other conditions that present with hepatosplenomegaly and ascites [10]. Critical differential diagnoses include pre-eclampsia or HELLP syndrome, both of which can cause liver dysfunction and abdominal pain [10,11]. Acute fatty liver disease of pregnancy (AFLP) is another critical consideration due to its association with liver failure and coagulopathy [11,12]. Portal vein thrombosis, liver cirrhosis, and right-sided heart failure can also present with similar signs of portal hypertension and ascites [10,11]. Additionally, infectious hepatitis and severe sepsis with liver dysfunction may mimic the clinical presentation of BCS [8-10]. Accurate differentiation from these conditions ensures timely and appropriate management [11,12]. Pregnancies complicated by BCS require close management, including adequate anticoagulation therapy, fetal growth monitoring, liver function assessment, and a multidisciplinary approach to care [8,9]. Yet diagnosing BCS remains challenging due to vague symptoms and rare occurrences, resulting in delayed clinical interventions [11-12].

This case shares significant parallels with several documented instances of BCS in pregnancy, where delayed diagnosis and management often led to poor outcomes. In a notable case report by Tian et al., a 29-year-old pregnant woman presented with symptoms of BCS in her third trimester, which was initially misinterpreted as preeclampsia [13]. Despite early signs of liver dysfunction and ascites, the diagnosis was delayed until severe hepatic decompensation ensued, leading to an emergency cesarean section [13]. Both the mother and fetus experienced significant complications, including postpartum hemorrhage and fetal distress, aligning with the adverse outcomes in our case [13].

Similarly, a report by Bendale et al. detailed the case of a 28-year-old woman diagnosed with BCS during her second pregnancy [14]. The diagnosis was complicated by overlapping symptoms of HELLP syndrome, as seen in our patient [14]. While anticoagulation therapy was initiated earlier in this case, fetal demise still occurred, illustrating the high maternal-fetal risk even with timely interventions [14]. In both Bendale’s case and our case, the presence of ascites, coagulopathy, and liver dysfunction created diagnostic confusion and delayed the identification of BCS [14].

Another case report by Oblitas et al. described a pregnant woman with BCS diagnosed in the postpartum period after developing sudden hepatic failure, ascites, and coagulopathy [15]. Despite aggressive treatment, the patient developed multiple complications, including sepsis, similar to our case, where rapid deterioration followed the cesarean section [15]. Both cases emphasize the necessity of early intervention and highlight how BCS can mimic other obstetric complications such as HELLP or severe preeclampsia [15].

Comparing these cases with ours, the common themes of diagnostic delay, overlapping symptoms with HELLP syndrome, and poor maternal-fetal outcomes emerge. What sets our case apart is the primigravida status of the patient, adding complexity to the management due to the lack of previous pregnancy history. The progression to intrauterine fetal death and liver failure in our patient further underscores the aggressive nature of BCS in pregnancy and the need for heightened clinical suspicion when faced with such presentations.

The lessons drawn from these cases emphasize that pregnant women with liver dysfunction, especially when complicated by ascites and coagulopathy, should undergo early Doppler ultrasonography or MRI to evaluate hepatic vein patency. The comparison to other case reports strengthens the argument for early intervention and individualized treatment plans, focusing on anticoagulation and multidisciplinary care to mitigate the severe complications of BCS in pregnancy.

## Conclusions

This case of BCS in a postpartum patient highlights the critical need for timely diagnosis and intervention. The patient's rapid deterioration despite initial management for suspected HELLP syndrome and sepsis underscores the importance of considering BCS in the differential diagnosis for postpartum women with persistent hepatic symptoms. Early recognition and a multidisciplinary approach are essential for improving outcomes in such complex cases. This report aligns with similar case studies, emphasizing the necessity for prompt and comprehensive management to address this rare but potentially life-threatening condition during pregnancy and postpartum.
